# Free dermatoplasty combined with vacuum sealing drainage for the treatment of large-area soft tissue defects accompanied by bone exposure in the lower leg

**DOI:** 10.3892/etm.2013.999

**Published:** 2013-03-12

**Authors:** JIAFU QU, RONGLIANG YAN, LIANG WANG, JUN WU, LIHAI CAO, GUOZHI ZHAO, KAIYAN SUN, LING ZHANG, XIAOJIAN DU, YI PENG, SHAOGUANG LI, HAIDONG MA, JIANHUA GAO, HONGDA LIU

**Affiliations:** 1 Department of Foot and Ankle Surgery, The Second Hospital of Tangshan, Foot and Ankle Surgery Institute of Tangshan;; 2 Department of Burn and Plastic Surgery, Tangshan People’s Hospital, Tangshan, Hebei 063000, P.R. China

**Keywords:** vacuum sealing drainage, lower leg, soft tissue trauma defect, bone exposure, free dermatoplasty

## Abstract

The aim of this study was to investigate the clinical efficacy of free dermatoplasty combined with vacuum sealing drainage (VSD) for the treatment of large-area soft tissue defects accompanied by bone exposure in the lower leg (*crus*). Free dermatoplasty combined with VSD was used to treat 36 patients with large-area soft tissue defects accompanied by bone exposure in the lower leg. The areas of the soft tissue defects ranged from 25×12 to 35×30 cm and the areas of exposed bone ranged from 6×4 to 10×6 cm. When evaluated by the open fracture Gustilo classification, 14 cases were of Gustilo type IIIA and 22 cases were of type IIIB. During surgery, adjacent available muscle flaps were transferred to cover the outer areas of the exposed bone and reduce the bone exposure range. Following VSD treatment, granulation tissues grew well and free dermatoplasty combined with VSD was used to treat and repair the wound surfaces. The patients were followed up for 1–5 years (mean duration, 2.5 years). All 36 cases with skin flap grafts survived, the free skin graft texture on the wound surface was good, the recovery of lower limb function was satisfactory and the success rate was 80.56%. Free dermatoplasty combined with VSD used for the treatment of large-area soft tissue defects accompanied by bone exposure in the lower leg may eliminate the need for amputation and complex surgery, and is a simple, fast and effective treatment method.

## Introduction

The number of patients with large-area soft tissue defects accompanied by serious injury involving bone exposure in the lower leg have increased due to rapid increases in road accidents and work-related injuries. In large-area skin soft tissue defects with poor blood supply and bone exposure, it is not feasible to conduct skin flap transplantation to close the wound and maintain the limb; therefore, amputation is often selected. Even if limb salvage treatment is selected, the traditional dressing therapy easily causes complications, including wound infection and non-healing, bone fracture and non-union, osteonecrosis, osteomyelitis and fistula formation. As a result, limb function is poor and the treatment efficacy is unsatisfactory. A novel vacuum sealing drainage (VSD) (1) technique has become the standard treatment method for treating various types of wound surfaces and wounds that are difficult to heal (2). Compared with other traditional drainage modes, it has clear advantages. VSD is an efficient drainage system and its efficiency embodies its comprehensive drainage and thorough drainage under high vacuum. It promptly and thoroughly leads seepage, pus and necrotic tissues from the drainage area out of the body to cause ‘zero accumulation’ in the drainage area. It also significantly speeds up infected lacuna closure and infected wound surface healing to effectively prevent surgical field effusion. Additionally, VSD is particularly suitable for the treatment of complex wounds. Experiments in animals suggest that the negative pressure environment promotes subcutaneous and intradermal blood flow in the wound, reduces bacterial reproduction and promotes granulation growth (3). Halvorson *et al*(4), by studying pediatric cases, confirmed that VSD is a safe and effective method with a low infection rate for treating open fractures. VSD combined with debridement and antibiotics increases the wound healing rate, shortens the hospitalization time and enhances patients’ comfort and satisfaction (5). To resolve the limb salvage problem, we used free dermatoplasty combined with VSD to treat 36 patients with large-area soft tissue defects accompanied by bone exposure in the lower leg from June 2006 to June 2011 and obtained a satisfactory efficacy.

## Subjects and methods

### General data

In this study, there were 36 patients, including 29 males and 7 females, aged 22–66 years with a mean age of 36.8 years. Among them, there were 14 cases whose left limb was injured, 19 cases whose right limb was injured and three cases with two injured limbs. Soft tissue defect areas ranged from 25×12 to 35×30 cm and bone exposure areas ranged from 6×4 to 10×6 cm. When evaluated by the open fracture Gustilo classification, 14 cases were of Gustilo type IIIA and 22 cases were of type IIIB. Regarding the cause of injury, 29 cases were due to traffic injury, five cases were due to crushing by heavy objects and two cases were due to wringer injury. The time interval from trauma to surgery was ∼4–10 h. In addition, emergency debridement was conducted in all cases. This study was conducted in accordance with the Declaration of Helsinki. and with approval from the Ethics Committee of the Second Hospital of Tangshan. Written informed consent was obtained from all participants.

### Treatment method

Emergency debridement was conducted under tourniquet control following epidural anesthesia or continuous epidural anesthesia. Wounds were repeatedly and thoroughly washed with a high pressure pulse flushing pump and sterilized with conventional disinfection solution. Debridement and hemostasis were strictly and thoroughly conducted to remove soft tissues without the loss of blood circulation. The initially denuded skins of the unclear necrosis boundary were maintained briefly. Once the necrosis boundary was clear, they were thoroughly removed. After stripping the skin and cleaning the subcutaneous fat, the fat was punctured with a knife to create meshed holes to enable the seepage of secreta, pus and necrotic tissues to drain away. Following emergency debridement, adjacent available muscle flaps were transferred to cover the outer areas of the exposed bone and reduce the bone exposure range. Several holes were drilled through the bare bone cortex surface into the contralateral cortex to enable blood capillaries and fresh granulation tissues to grow from the channels to cover the exposed bone. In this study, all cases were treated by VSD. After 1–4 rounds of treatment, granulation tissues on the wound surface grew well and free dermatoplasty was conducted to close the wound. Following the free dermatoplasty, sustained low-pressure VSD was conducted. In the donor skin area, sustained VSD was conducted for 12 cases. The wound surface was firstly covered with Vaseline gauze and then covered with VSD dressing. For situations involving open fractures, external fixing frame fixation or simple fixation, including Kirschner wire and screw fixation, was used.

When applying a sterile medical sponge (VSD dressing) to the wound surface, it is appropriate to cover only the wound surface. A transparent adhesive membrane (semipermeable membrane) was then used to seal the wound and the VSD dressing. The semipermeable membrane extended >3 cm from the edge of wound to seal the surrounding normal skin. Thus, the open wound was closed. A silicone tube was connected between the VSD dressing and a VSD special treatment instrument or a negative pressure drainage device, and negative pressure treatment was conducted in a closed condition. A study has confirmed that a negative pressure maintained at 125 mmHg increases microcirculation while a negative pressure >400 mmHg causes retroaction (6). According to the particular situation of each wound, either intermittent or sustained constant negative pressure treatment was selected. When the wound surface was relatively small, the amounts of seepage and secretions were less and the wound surface was cleaner and an intermittent constant negative pressure was selected only when it was required to rapidly promote the growth of granulation tissues. When the wound surface was larger and the amounts of seepage and secreta were greater, a sustained constant negative pressure treatment was selected in order to promptly and effectively lead waste out of the body. Generally, the VSD dressing and semipermeable membrane were replaced once every ∼7 days. When there was a substantial amount of waste, including pus, secreta and necrotic tissues, drainage tubes in the VSD dressing or VSD dressing micropores became obstructed, which affected the filtering and suction of the VSD dressing. In these conditions, the VSD dressing and semipermeable membrane were replaced once every 3–4 days. During treatment, it was necessary to maintain a sealed environment and an effective negative pressure status, as well as unobstructed pipelines.

### Typical case 1

**Figure 1:**
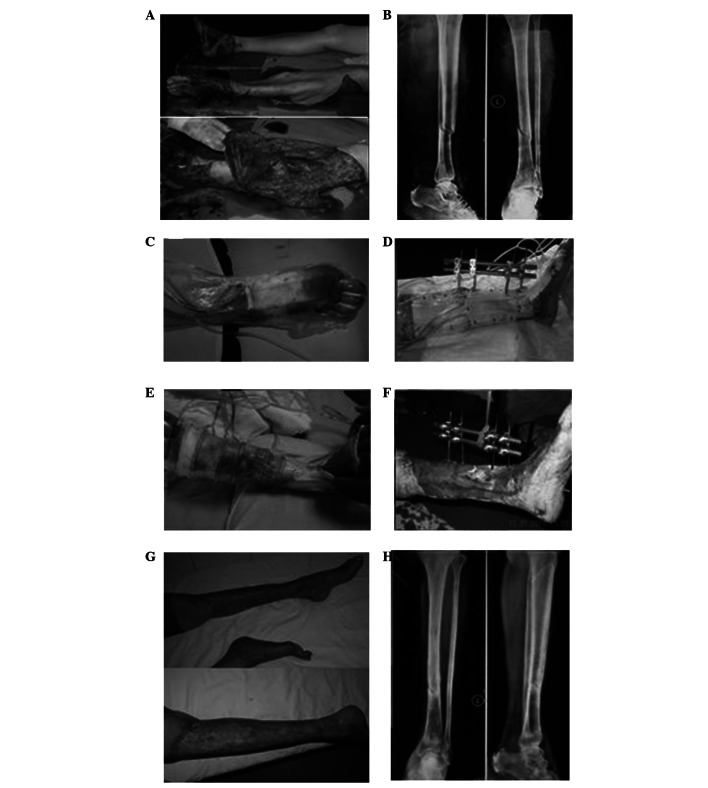
(A) Left lower leg and left foot traumas (Gustilo type IIIB), right ankle trauma (Gustilo type IIIB); (B) X-ray revealed a left tibial fracture; (C) once the right foot wound was debrided, vacuum sealing drainage (VSD) was conducted; (D) external fixing frame fixation combined with VSD was conducted for the left tibial fracture; (E) after three VSD treatments, granulation tissues grew well on the lateral wound in the lower leg; (F) an exposed bone area of 6×4 cm remained in the medial area; (G) the wound healed well at 8 weeks following free dermatoplasty; (H) at 12 weeks after tibial fracture, the fracture line was indistinct.

Case 1 was a female patient aged 60 years with traumatic hemorrhagic shock, left lower leg and left foot traumas (Gustilo type IIIB) and right ankle trauma (Gustilo type IIIB). After VSD had been conducted on the right foot for 3 weeks, free dermatoplasty was conducted on the fresh granulation tissues on the wound surface and sustained low-pressure VSD treatment was conducted in the skin transplantation area. After 1 week, the skin graft had completely survived and the wound was healed. In addition, after VSD treatment had been conducted on the left lower leg and foot for 3 weeks, local muscle flap transfer was conducted to cover the exposed tibia. After 1 week, the muscle flaps had survived and free dermatoplasty was conducted. In the skin tranplantation area, sustained low-pressure VSD treatment was conducted. After 1 week, the skin graft had completely survived and the wound had healed well (Fig. 1).

### Typical case 2

**Figure 2:**
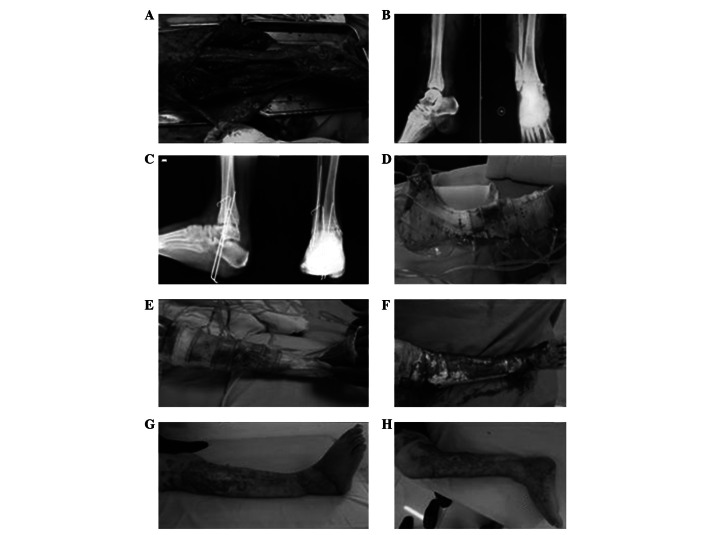
(A) Open fracture dislocation of the right lower extremity (Gustilo type IIIC); the area of exposed bone was 10×5 cm; (B) X-ray revealed open, comminuted fractures of the right internal and external ankles and open dislocation of the right ankle joint; (C) internal fixation with a Kirschner wire was conducted for the open fracture dislocation; (D) following wound debridement, vacuum sealing drainage (VSD) was conducted; (E) after wound debridement was conducted again, VSD treatment was conducted; (F) the wound following three treatments of VSD; (G and H) 12 weeks after free dermatoplasty, the wound was well healed; (G) front view; (H) lateral view.

Case 2 was a male patient aged 34 years, with an open fracture dislocation of the right lower extremity (Gustilo type IIIB). After two VSD treatments, local muscle flap transfer was conducted to cover the exposed tibia. After 1 week, the muscle flaps had survived and free dermatoplasty was conducted. In the skin tranplantation area, sustained low-pressure VSD treatment was conducted. After 1 week, the skin graft had completely survived and the wound had healed well (Fig. 2).

## Results

### Treatment results

Among the 36 patients, VSD was conducted 1–4 times according to the status of the wounds and the degree of bone exposure (larger bone exposure meant a greater frequency of VSD). Among them, seven cases of soft tissue injuries were mild and the bone exposure area was 6×4 cm. In the 6–14 days following the first treatment with VSD, the wound surfaces were completely covered by granulation tissues. In 12 cases of bone exposure, the area ranged from 7×4 to 57×5 cm. At days 13–24, following two VSD treatments, the wound surfaces were completely covered by granulation tissues. In nine cases of bone exposure, the area ranged from 8×4 to 8×5 cm. At days 25–27, following three VSD treatments, the wound surfaces were completely covered by granulation tissues. In eight cases of bone exposure, the area ranged from 9×5 to 10×5 cm. At days 26–39, following four VSD treatments, the wound surfaces were completely covered by granulation tissues. Following emergency debridement, the exposed bone was covered with adjacent muscle flaps. Following VSD treatment, abundant, fresh and actively bleeding granulation tissues with good elasticity grew on the wound surface. In stage II, the exposed bone was covered with adjacent muscle flaps in 12 cases and the exposed bone was covered with rapidly growing granulation tissues. The time taken for the exposed bone to be completely covered in the 36 patients ranged from 6 to 29 days and the mean was 18.2 days. Once free dermatoplasty combined with sustained low-pressure VSD was conducted, 36 cases of skin flap grafts survived. Among them, stamp skin transplantation was conducted for five cases for 6–8 days due to the larger free skin transplantation area and the limited donor skin area. Once the VSD dressing had been removed, scattered wound areas remained in gaps of the skin graft. Dressings were actively administered and the wound surfaces healed. Fracture healing durations ranged from 3 to 6 months and the mean duration was 4 months. No osteonecrosis and no osteomyelitis occurred. In addition, three cases suffered from achilles tendon contracture of equinus deformity and diorthosis was conducted.

### Postoperative follow-up

The cases in this study were followed up for 1–5 years (mean duration, 2.5 years). The textures of the free skin grafts on the wound surfaces were good and recovery of lower leg and ankle functions were satisfactory. Lower leg and ankle functions were scored according to the Iowa evaluation rating system criteria (7): excellent, eight cases; good, 21 cases; qualified, five cases and poor, two cases. The rate of excellent or good functional results was 80.56%.

## Discussion

VSD reduces wound infection and promotes wound healing. Following the debridement of large-area soft tissue defects accompanied by bone exposure in the lower leg, VSD is conducted to transform the open wound into a closed wound and prevent external bacteria from invading the wound surface. Therefore, it eliminates the conditions that favor bacterial culture, inhibits bacterial growth and reproduction, blocks infection spread and toxin absorption, decreases bacterial infection and reproduction levels in the tissue, decreases the wound infection rate, helps the rapid growth of granulation tissues on the wound surface and promotes wound healing. Experimental and clinical studies have confirmed that the negative pressure-assisted wound surface closing technique effectively promotes wound surface healing by multiple mechanisms (8). The closed environment and continuous negative pressure suction of VSD cause the wound surface to form a sustained hypoxic or relatively anoxic subacid environment and thus inhibits the growth of pathogenic microorganisms on the wound surface. Additionally, VSD causes a drop in the oxygen tension around the wound surface to stimulate initiation of the repair signal. Therefore, the release of fibrinolytic activator and other enzymes is promoted to form an environment of quickening fibrinolysis. Subsequently, fibrinolysis occurs on the wound surface and autolytic debridement is conducted (9), which contributes to wound surface healing. VSD promotes cellular proliferation and migration (10). Labler *et al*(8) confirmed that following VSD treatment, the levels of interleukin-8 and vascular endothelial growth factor expression on the wound surface were significantly increased, which promoted vascularization of the wound surface. Morykwas *et al*(11) reported that VSD treatment accelerates the blood circulation on the wound surface and that the intermittent peak in the soft tissue blood supply flow was 4-fold higher than the normal baseline blood flow. The treatment of this group of cases also confirmed that VSD enables the soft tissue to obtain a good blood supply and rapidly relieves swelling of injured tissue, reduces local edema, improves local circulation and oxygen level, speeds up the growth of fresh granulation tissues and promotes wound healing. The treatment of complex wounds often requires multiple debridements and the wounds are finally closed by skin flaps or skin grafts. VSD helps to shorten the treatment time and simplifies the treatment method (12).

Large-area soft tissue defects accompanied by bone exposure in the lower leg are difficult to treat. Due to the large area of the skin soft tissue defects, poor blood supply and bone exposure, it is not feasible to use skin flap transplantation to close the wound surface. Additionally, the defects are often accompanied by surrounding infection and osteonecrosis, as well as osteomyelitis. Therefore, it is difficult to maintain the limb and amputation is the only available option. Even if limb salvage treatment is selected, with traditional dressing therapy the drainage of seepage, pus and necrotic tissues is poor, which causes the wound surface to become a bacterial culture medium. Bacterial growth and reproduction are likely to cause complications, including wound infection and non-healing, bone fracture and non-union, osteonecrosis, osteomyelitis and fistula formation. As a result, limb function is poor and treatment efficacy is unsatisfactory. Free dermatoplasty combined with VSD is an effective treatment method. Li *et al*(13) used VSD to treat large-area soft tissue defects in child lower leg and identified that VSD effectively prevents exposed deep soft tissue infection, and granulation tissues grew well around exposed bones and tendons. The early surgical treatment principle of large-area soft tissue defects accompanied by bone exposure in the lower leg resolves soft tissue coverage and healing problems on the wound surface. Prior to surgery, it is necessary to evaluate the status of the soft tissue injury to guide the treatment and judge the prognosis. For all cases in this study, several holes were drilled through the bare bone cortex surface into the contralateral cortex. Following VSD treatment, fresh granulation tissues grew from the channels and an adjacent muscle flap transfer was conducted to cover the bone surface. The time taken for the bone to be completely covered ranged from 6 to 29 days and the mean was 18.2 days. Therefore, a good soft tissue base was provided for free skin graft reparation of the wound surface. For all cases in this study, free dermatoplasty of transferred adjacent muscle flaps combined with VSD was used for treatment. It avoids amputation and complex surgery, solves the problem of the traditional dressing method by allowing thorough drainage, promotes the rapid growth of granulation tissues and speeds up wound healing. Mouës *et al*(14) compared the efficacies of local negative pressure therapy and traditional closing treatment of the wound surface of serious injuries in a prospective randomized study, and identified that in the negative pressure therapy group, the healing of the wound surface occurred earlier and the complication occurrence rate was lower. In the current study, three cases suffered from achilles tendon contracture of equinus deformity (diorthosis was conducted) and in seven cases, the functional evaluations were relatively poor (qualified, five cases; poor, two cases) due to severe injuries, a number of soft tissue defects around the lower leg, lack of muscular strength and flexibility and bone paste scar formation. However, the patients themselves felt that the result was better than wearing a prosthesis. As plantar skin soft tissue injuries are milder, there are normally no severe injuries; therefore, patients are able to apply a load and walk.

The current study confirms that the application of VSD combined with free dermatoplasty of transferred muscle flaps in large-area soft tissue defects accompanied by bone exposure in the lower leg avoids amputation and complex surgery, greatly shortens the treatment time, speeds up wound healing, significantly reduces complications, markedly decreases the infection rate and clearly increases the treatment success rate. It is a simple, fast and effective treatment method. Hou *et al*(15) suggested that VSD treatment of a tibial Gustilo type IIIB fracture may reduce the skin graft or free skin flap area. The retrospective study conducted by Babiak *et al*(16) suggested that VSD shortened the treatment time.

For large-area soft tissue defects accompanied by bone exposure in the lower leg, it is inappropriate to conduct adjacent flap transfer and contralateral cross leg flap transfer to close the wound surface and more appropriate to conduct skin flap transplantation to close the wound, due to the large area of the soft tissue defect, the severity of the soft tissue injury and poor vascular conditions. A good soft tissue base bed and healthy fresh granulation tissues are required for free skin graft reparation of the wound surface. For exposed bone, adjacent muscle flap transfer is conducted to cover the outer areas of the exposed bone and reduce the bone exposure range. In the current study, once the soft tissue defect areas had been treated by VSD, abundant, fresh and active-bleeding granulation tissues with good elasticity grew on the wound surface, which provided a good soft tissue bed for free skin graft reparation of the wound surface. Large-area free skin flaps are preferred and skin flaps are connected by suturing. Stamp skin grafts or punctuate skin grafts are not used if possible in order to reduce scarring and the repeated damage generated by granulation tissue proliferation in graft gaps. Large-area free skin grafts in the lower leg are inappropriately compressed by packing. One reason lies in the large area of the wound surface as it is impossible to apply a compression bandage uniformly. Another reason lies the pressure not being evenly controlled, which is likely to affect the venous return of the limb. Webster *et al*(17) confirmed that VSD reduces the transplantation failure rate. Although the daily cost of VSD is higher, VSD reduces dressing requirements and promotes wound healing and thus reduces hospitalization time (18). For all cases in this study, sustained VSD treatment was conducted following free dermatoplasty. Clinical studies confirm that VSD causes a mechanical reaction to negative pressure but does not readily move skin flaps. Furthermore, it removes surplus fluid, reduces infection, speeds up wound vascularization and promotes the rapid growth and healing of free skin grafts.

The following matters require attention during VSD: i) it is important to conduct thorough debridement of wounds to remove all inactive tissues, since necrotic tissues act as bacterial reproduction foci and resolvase and bacteriotoxin are factors that hinder wound healing. ii) The wound range must be reduced as far as possible. Available adjacent muscle flaps are transferred to cover the outer areas of the exposed bone to reduce the bone exposure range. Exposed tendon and bone surfaces are covered with a layer of medical foam sponge to avoid cavities on the wound surfaces. iii) VSD treatment should comply with surgical procedures. It is necessary to maintain a sealed, effective and negative pressure status and maintain unobstructed pipelines. Retrograde flushing should not be conducted. iv) The silicone tube inserted for intermittent flushing during surgery should be maintained in a closed status during the on-flushing period. v) During treatment, peripheral blood circulation of the toes and the patient’s vital signs should be observed to prevent negative nitrogen balance. As the removed exudant contains large amounts of proteins, it is necessary to supplement adequate nutrients. At the beginning of VSD treatment, individual patients may present wound pain and it is feasible to apply analgesics or reduce the negative pressure. With an increase in tolerance, the pressure may be adjusted gradually to the required negative pressure status. If wound secretions increase, they are removed by sustained high negative pressure. vii) Active hemorrhaging in the drainage area is a contraindication of VSD treatment.

VSD dressing is a non-absorbable material and the material itself does not provide nutritional elements for the exposed tendons and bones on wound surfaces. The development of biological products into VSD dressings providing nutritional elements for exposed tendons and bones on wound surfaces, for fresh granulation tissues rapidly growing on the surfaces of exposed tendons and bones and for preventing dry necrosis of exposed tendons and bones, it is likely to be a new milepost in the development of VSD and should be further investigated.
